# Safety and Feasibility of Low Fluence Intense Pulsed Light for Treating Pediatric Patients with Moderate-to-Severe Blepharitis

**DOI:** 10.3390/jcm11113080

**Published:** 2022-05-30

**Authors:** Zimeng Zhai, Hao Jiang, Yuqing Wu, Pei Yang, Shuyun Zhou, Jiaxu Hong

**Affiliations:** 1 Department of Ophthalmology and Visual Science, Eye and ENT Hospital, Shanghai Medical College, Fudan University, 83 Fenyang Road, Shanghai 200031, China; zimeng_zhai@163.com (Z.Z.); kidd5jh@sina.com (H.J.); yokchingwu@163.com (Y.W.); pei_28@163.com (P.Y.); zhoushuyun@sina.com (S.Z.); 2 Department of Ophthalmology, The Affiliated Hospital of Guizhou Medical University, Guiyang 550004, China

**Keywords:** blepharitis, children, intense pulsed light, meibomian glands

## Abstract

To explore the safety and feasibility of low fluence intense pulsed light (IPL) for treating pediatric patients with moderate-to-severe blepharitis and to analyze potential factors associated with the recovery of meibomian glands (MG) dropout, a retrospective, noncomparative study, including 17 blepharitis patients (33 eyes) aged between 5 and 16 years old was conducted. All of the participants were given 4 continuous sessions of low-fluence (9–12 J/cm^2^) IPL at 3–4 week intervals. Corneal fluorescein staining (CFS), tear breakup time (BUT), inferior tear meniscus height, Demodex presence, and MG morphology were examined before and after the treatment. Results indicated that CFS, BUT and MG morphology (central/total gland area ratio and gland signal index) had significantly improved (*p* < 0.05). Symptoms and signs such as severe corneal neovascularization, limbal pannus and conjunctival congestion also subsided. Among age, gender, presence of Demodex and interval before diagnosis, age initiating the formal treatment was confirmed as a negatively correlated factor of the recovery of MG dropout (*p* = 0.032, B = −1.755). No notable adverse events were reported. In conclusion, low fluence IPL seems to be a safe and effective alternative for moderate-to-severe pediatric blepharitis, and MG dropout is prone to recover in younger patients.

## 1. Introduction

Blepharitis is an ocular inflammatory status that primarily involves the eyelid margin, causing recurrent hordeolum/chalazion and irritation symptoms such as itchiness, redness, tearing and burning sensation of the ocular surface. The patient population is distributed across all ages, affecting up to 47% patients in the clinical practice of optometrists [1]. By contrast, the degree of concern for blepharitis patients has been low, especially for children [2]. Similarly to adults, their clinical signs and symptoms are also graded as mild, moderate, or severe [3]. In pediatric cases, secondary conjunctival and corneal involvement after blepharitis, namely blepharokeratoconjunctivitis (BKC), is more frequent, such as punctate keratopathy, corneal neovascularization, even corneal scarring and induced astigmatism when delayed in diagnosis [4]. Opportune detection and treatment of blepharitis can reduce the clinical symptoms and permanent structural damage. The general management for blepharitis among children includes eyelid hygiene, warm compresses, topical and/or systemic antibiotics, topical anti-inflammatory agents, or a combination thereof [3]. However, some methods can cause great discomfort, such as eyelid massage and topical cyclosporine drops, which hardly gets full cooperation from both children and parents. Topical antibiotics/steroids are common usage for the treatment of BKC [5,6]. Nevertheless, the elevation of intraocular pressure and other side effects of steroids restrict their further applications [7]. Oral azithromycin combined with topical anti-inflammatory agents have showed positive results in a small series of BKC children, however, there is no valid evidence supporting the use of systemic antibiotics for moderate-to-severe pediatric blepharitis [8,9]. Given their gastrointestinal side-effects and risk of hearing impairment with azithromycin, reduced dose and the shorter course of therapy should be adopted [10]. A safe and effective alternation is imperative considering the chronic nature of blepharitis, especially in susceptible pediatric population.

Intense pulsed light (IPL) therapy has been studied in adults to improve meibomian gland (MG) secretion, reduce eyelid telangiectasias, and eradicate blepharitic Demodex mainly through the photothermal effect [11]. From toddlers to adolescents, IPL has been frequently employed in the dermatology field concerning the removal of facial hair and port-wine birthmark, the impediment of scar formation, with no remarkable side effects documented [12,13,14,15]. However, few published articles have discussed the feasibility of IPL in pediatric blepharitis. In a case report of a 10-year-old, 12.2 J/cm^2^ IPL could effectively shorten his BKC course within 17 days [16]. Different from the common fluence levels of 12–16 J/cm^2^ for adults, children were reported to be vulnerable to light therapy [17]. Thus, we decided to use low fluence IPL on our patients to reduce the possible adverse effects. Herein, the current observational study was to evaluate the safety and feasibility of IPL for pediatric populations with moderate-to-severe blepharitis and to explore the changes to their MGs.

## 2. Methods

### 2.1. Study Participants

We retrospectively analyzed blepharitic patients aged between 5 and 16 who underwent IPL therapy at Eye and ENT Hospital of Fudan University from February 2019 to October 2021. This research followed the tenets of the Declaration of Helsinki and was approved by the Ethics Committee of Eye and ENT Hospital of Fudan University (EENTIRB-20190301). Before the IPL treatment, informed consent was obtained from all of the participants’ legal guardians.

The inclusion criteria for this study were listed as follows: (1) between the age of 5 and 16; (2) Fitzpatrick skin types I–IV; (3) presented with at least one moderate or severe symptom, for example, recurrent chalazion, irritation symptoms, foreign body sensation, or blurred vision; (4) presented with at least one moderate or severe clinical sign, for example, eyelid telangiectasias, eyelash scales, conjunctival congestion, corneal neovascularization, or ocular surface staining; (5) maximal medical therapy failure; (6) able to comply with all the treatments and follow-up visits according to the schedule. The exclusion criteria for IPL therapy were as follows: (1) acute inflammation; (2) abnormal eyelid structure, such as eyelid defect, entropion or ectropion; (3) co-occurring with additional eye disorders or trauma; (4) ocular affected systemic immune diseases, such as Sjogren’s syndrome, rheumatism, and hyperthyroidism; (5) severe systemic diseases, such as cardiovascular or central nerve diseases; (6) active facial skin lesions; (7) previous laser or light-based therapies within 1 month; (8) other conditions judged by the researchers as unsuitable for this study.

### 2.2. Clinical Assessments

The clinical assessments of the enrolled participants included corneal fluorescein staining (CFS), tear break-up time (BUT), inferior tear meniscus height (TMH), Demodex presence, and MG morphology. The Ocular Surface Disease Index questionnaire was only suitable for patients who can respond adequately and not included in our assessment indexes. Subjective symptoms were mainly acquired by guardians’ dictation. CFS was evaluated during slit-lamp examination under cobalt blue illumination. After a drop of fluorescein sodium, superficial punctate keratopathy of the cornea would be scored between 0 and 3 in five areas (upper, lower, nasal, temporal, and central zone) then summed up for analysis (maximum score of 15). BUT, TMH, and MG morphology were obtained via a noncontact ocular analyzer Keratograph 5M (OCULUS Optikgeräte GmbH, Wetzlar, Germany), according to the published studies [18,19]. BUT and TMH data was collected through infrared photographs of the anterior segment captured by the analyzer. Participants were asked to blink twice and then keep their eyes wild open for as long as possible. The following blink automatically terminated the measuring process, during which period the area and timepoint of tear film rupture should be recorded by the equipment. BUT was interpreted as the first break-up time of the tear film in this study. TMH represented the length of inferior tear meniscus perpendicular to the lower eyelid margin, which also could be measured by the Keratograph analyzer. MG morphology was observed through the infrared images of everted upper and lower eyelids. The array of “string-like” structure was defined as MGs and could be profiled with an automatic and multiparametric algorithm (Meibomian Gland Bioimage Analyzer, ZOC-CODE-JY-1) [20]. This verified algorithm was designed to perform repeatable analysis of meibography images and to provide the central/total gland area ratio (%), gland tortuosity index to quantify the degree of gland curving, and gland signal index to evaluate the meibum secretion. Partial loss or truncation of MGs was regarded as MG dropout. Demodex was carefully examined by 6~8 freshly removed eyelashes under an optical microscope. An interval of 5 min was required between each examination to minimize the interference. All the exams above were conducted before our therapy (baseline) and 3–4 weeks after the last treatment by the same researcher.

### 2.3. Treatment

**Figure 1:**
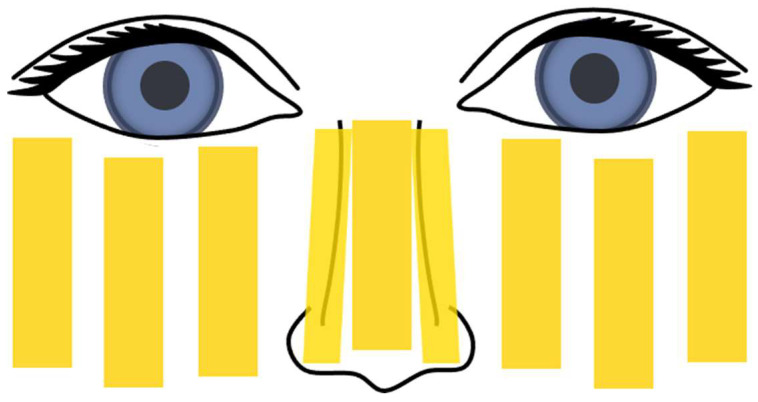
IPL treatment sites (marked in yellow). Approximately 9 symmetrical pulses were applied on the nose and cheek.

All of the participants discontinued at least 3 weeks of ineffective medical therapy but previous topical Moxifloxacin and eye atomization used as mild adjuvant methods. A complete course consisted of four IPL sessions at 3–4 weeks interval, with a total duration of 3 months. Eye drops were used 3 times a day. Ultrasonic nebulizer (Jiangsu Yuyue Medical Instruments Co., Ltd., Yancheng, China) was filled with 0.3% hyaluronic acid sodium eye drops and 0.9% normal saline for 20 min of binocular atomization. IPL (SOLARI, Lutronic Corporation, Goyang, Korea) was used at filter of 570 nm, fluence of 6–9 J/cm^2^, three-pulse mode (pulse width of 6 ms, pulse delay of 50 ms) according to the age and skin type of participants. After placing the eye shields and cooling gel, approximately 9 overlapping pulses were applied below the lower eyelid, as shown in Figure 1. MG expression was rescinded because of the unbearable pain for children. Final evaluations were performed by global assessment and patient satisfaction 3–4 weeks after the last treatment of a consecutive course of IPL therapy, from which their further regimen was determined, including the cessation of all medical means, another integral IPL course or switching back to medications such as oral azithromycin (15 mg/kg/daily) and 0.05% cyclosporine drops (tid). Adverse effects were monitored at each treatment session and follow-up visit.

### 2.4. Statistical Analysis

Data were analyzed using SPSS 20.0 (IBM Corp., Armonk, NY, USA) for statistical analysis. The descriptive data were described as the Mean and Standard Deviation (SD). Due to the distinctive risk of impeding visual development for children, our participants with blepharitis were all recommended to accept IPL therapy by the researchers. For comparisons of pre- and post-treatment, normally distributed continuous variables were analyzed using paired t-test and non-normally distributed ones were analyzed using nonparametric Wilcoxon signed-rank test. Single-factor analysis was used to test for the possible association between the therapeutic effect of IPL and the most intuitive parameters of child patients (age, gender, interval before diagnosis, and presence of Demodex) for purposeful usage of IPL in future practice. Subsequently, a multivariate analysis was performed including all parameters that were possibly associated (*p* < 0.20) to adjust the underlying correlation between assessed parameters. *p* < 0.05 was considered statistically significant for all analyses.

## 3. Results

### 3.1. Patients’ Demography

A total of 33 eyes of 17 patients with blepharitis were included in our retrospective study, which consisted of 7 males and 10 females with a median age of 12.3 ± 3.6 years (range of 5–16 years). The Fitzpatrick skin types were III or IV. Before receiving the professional treatment from the Ophthalmology Department of Eye and ENT Hospital of Fudan University, participants had an average interval before diagnosis of 16.9 ± 15.1 months (range of 1–48 months). The detailed information of participants is displayed in Supplementary Materials.

### 3.2. Safety and Efficiency Assessment

**Figure 2:**
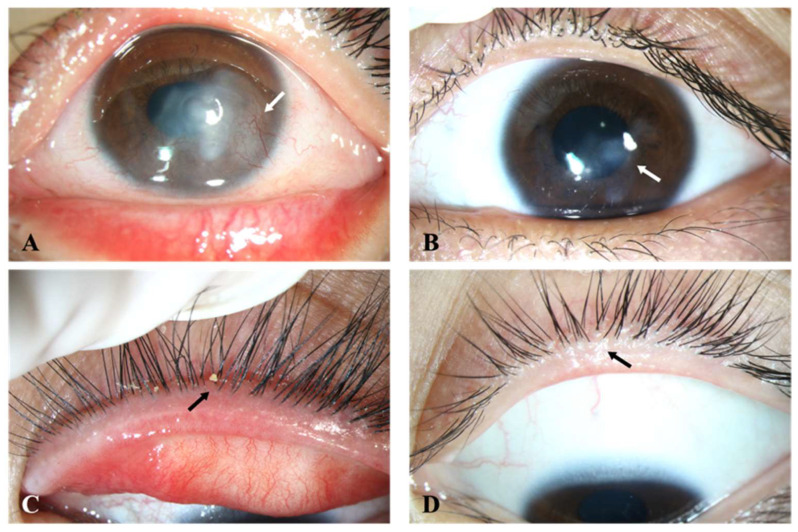
Changes in ocular signs before and after IPL treatment. (**A**,**B**) Severe corneal neovascularization (white arrow), conjunctival congestion and (**C**,**D**) eyelash scales (black arrow) had a marked improvement.

**Figure 3:**
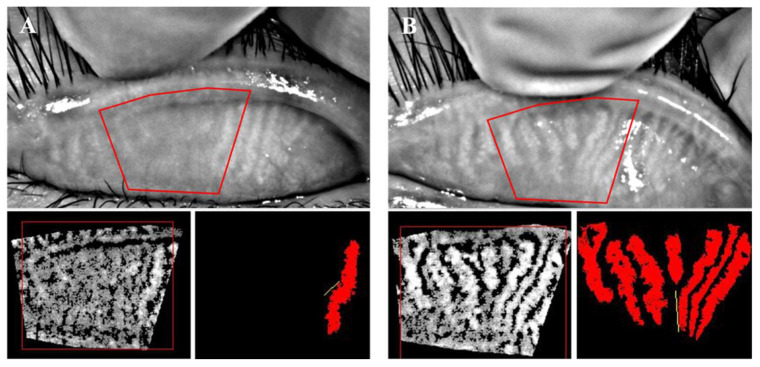
A representative case of meibomian gland (MG) recovery: (**A**) MG morphology before the IPL treatment; (**B**) The same MG morphology after a continuous course of IPL treatment. The central MGs in red frame had an obvious reappearance.

After IPL treatment, the clinical symptoms and signs of participants were all on the mend to varying degrees. Severe corneal neovascularization, limbal pannus, eyelash scales and conjunctival congestion had subsided after a course of treatment (Figure 2). Table 1 shows the ocular-surface associated indexes of the IPL group before and after treatment. In the baseline examination, 14 out of 33 eyes were CFS positive whereas 8 remained positive at the end of our study, among which the collective staining score was significantly reduced (*p* = 0.003). BUT had a notable improvement after the therapy compared with the baseline (*p* = 0.004). There was no significant change in the average TMH. Furthermore, the central and total gland area ratio both achieved a statistically significant increase from the pretreatment status (*p* = 0.003 and 0.026, respectively). The gland signal index, namely the lipid content of MGs also significantly increased (*p* < 0.001). There was no statistical difference in the tortuosity of MGs (*p* > 0.05). Figure 3 showed representative changes in MG morphology. All of the participants reported no moderate or severe adverse events.

### 3.3. Factors Associated with the Recovery of MG Dropout

Upon an initial single-factor analysis, age (*p* = 0.022) and interval before diagnosis (*p* = 0.132) were listed as suspicious factors related to the amelioration of MG area ratio. The subsequent multivariate analysis of these non-independent factors confirmed the age initiating the formal treatment as a negatively correlated factor (*p* = 0.032, B = −1.755). Detailed information between the improvement of MG recovery and other variables are shown in Table 2.

## 4. Discussion

Blepharitis represents an obstinate issue to normal activities for both adult and child patients. However, the relative scarcity of powerful treatments available for pediatric blepharitis makes the vision-threatening disease more prominent. As a common form of therapy in dermatology, IPL has been constantly attempted in child population for difficult miscellaneous diseases with no severe adverse events reported. Therefore, to clarify the safety and effects of IPL on moderate-to-severe children blepharitis, we conducted a series of ocular surface-related examinations after exposing to IPL and compared them with the baseline results of our participants.

Our study confirmed a notable improvement of symptoms, CSF and BUT in pediatric blepharitis after 4 consecutive IPL treatments, which was in accordance with the effects of IPL on adults [21,22,23]. What intrigued us most was the remarkable change in the MG morphology after IPL treatment. The MG proportion, no matter the central 5–8 glands or total glands in the eyelids, had undergone a notable morphological recovery from the pretreatment status. Recently, Yang et al. discovered that human MG epithelial cells could de-differentiate into proliferating cells upon exposure to appropriate environmental stimuli [24]. This provides a solid theoretical basis for the possible regeneration of MGs. In 2018, Gong and her group reported that MG microstructure and positive rate of inflammatory cells were improved under IPL treatment in adults [25]. They surmised, therefore, that the very improvement of MGs was induced by the photomodulation and anti-inflammatory effect of IPL. Photomodulation was the photobiostimulatory effect originally developed for a NASA space experiment, possessing the ability to promote cell activity and wound healing, which might modulate the regeneration of MGs [26]. One key mechanism of anti-inflammation effect of IPL is to cause the localized destruction in abnormal blood vessels (thrombosis), reducing an important reservoir of inflammatory mediators [11]. This might explain the relief of eyelid telangiectasia, conjunctiva congestion and even corneal pannus in our study. Given the positive correlation between patient CSF, BUT and MG lipid content, it is strongly suggested that the increased lipids drained into the tear film have sustained the BUT, thus alleviating the CFS during the course of IPL. We then speculated that IPL not only improves the MG morphology, but also gland function via producing and secreting more lipids into the tear film of our blepharitis patients. Moreover, according to the multivariate analysis, age, when initiating the formal treatment, was negatively correlated with the degree of MG morphology improvement, which strongly indicated that younger patients hold higher potential of MG recovery under IPL treatment. This study provided primary evidence supporting the above hypothesis.

No remarkable adverse events such as erythema or pigmentary changes were detected in the current study. The uncooperative behavior before MG photography or IPL could be eliminated by gentle conciliation. All of the participants (5–16 years old) were able to tolerate IPL treatment without complaining of pain or heat. This might be attributed to the low IPL fluence used on our participants. For refractory dermatosis, the fluence level of IPL could reach as high as 18–22 J/cm^2^ in pediatric patients [15]. In a case report of a 10-year-old, IPL of 12.2 J/cm^2^ could greatly ease the BKC symptoms [16]. However, another retrospective study described a transient headache on 3 pediatric BKC patients the night of IPL at 9–13 J/cm^2^ [17]. They speculated that children’s skin was more sensitive to light and that the presence of parasympathetic nerves in the auriculotemporal region might lead to headache symptoms at night. In our study, the fluence level of IPL was 6–9 J/cm^2^ and no adverse effects was observed in all 17 participants. It might be suggested that IPL therapy with lower fluence level is more congruent with pediatric blepharitis or even facial-laser-indicated skin disorders. One limitation of this observational study is the lack of control group, which stems from the discrepancy of treatment program between our moderate-to-severe pediatric cases and other mild or adult blepharitis. Another limitation is the small sample size of this study and subsequent difficulty learning the optimized IPL fluence for children. Further cytological and molecular tests are requisite to fully elucidate the mechanisms in IPL treating child blepharitis. 

In conclusion, low fluence IPL could effectively improve the symptoms and signs of pediatric blepharitis without notable adverse effects, and younger population might possess higher potential for MG recovery under IPL treatment. This study offered a prospective adjuvant treatment with low side-effect profile and high efficacy on refractory pediatric blepharitis. Research with larger samples and a longer observation period is needed to draw further conclusions.

## Figures and Tables

**Table 1:** Patient data of ocular-surface indexes before and after treatment.

		Pre-Treatment	Post-Treatment	*p* Value
CFS		1.42 ± 2.45	0.45 ± 1.03	0.003 *
BUT (s)		5.59 ± 3.76	7.37 ± 3.20	0.004 *
TMH (mm)		0.20 ± 0.07	0.21 ± 0.06	0.081
MG Morphology	CR (%)	45.61 ± 13.22	51.66 ± 8.56	0.003 *
	TR (%)	70.66 ± 17.24	76.48 ± 8.29	0.026 *
	GT	8.81 ± 3.09	9.82 ± 3.86	0.119
	GS	5.36 ± 1.43	6.10 ± 1.63	<0.001 *

CFS, corneal fluorescein staining; BUT, tear break-up time; TMH, inferior tear meniscus height; MG, meibomian gland; CR, central gland area ratio; TR, total gland area ratio; GT, gland tortuosity index; GS, gland signal index. * *p* < 0.05 was considered statistically significant.

**Table 2:** Correlation Between the Level of Meibomian Gland Recovery and Other Variables.

Single-Factor Analysis	Age	Gender	Interval before Diagnosis	Demodex
R	−0.397	−0.164	−0.268	−0.134
*p*	0.022 *	0.362	0.132 †	0.458
Logic regression analysis of the correlation between the suspicious variables and meibomian gland recovery				
**Predictor**	**B**		**SE**	***p***
Age	−1.755		0.816	0.032 *
Interval before diagnosis	−0.176		0.801	0.827

* *p* < 0.05; † suspicious variable related to meibomian gland recovery. SE, standard error.

## Data Availability

The data supporting the reported results are available from the corresponding author upon reasonable request.

## Supplementary Materials

The following supporting information can be downloaded at: https://www.mdpi.com/article/10.3390/jcm11113080/s1, Table S1: Detailed patient characteristics and clinical data of ocular-surface indexes.
