# Supplementary material for: Safety and Feasibility of Low Fluence Intense Pulsed Light for Treating Pediatric Patients with Moderate-to-Severe Blepharitis
Source: J Clin Med. 2022 May 30;11(11):3080. doi: 10.3390/jcm11113080 (PMC9181558; doi:10.3390/jcm11113080)
Supplement: Supplementary file 1 [file jcm-11-03080-s001.zip › jcm-1644066-supplementary.pdf]

Supplementary Table S1. Detailed patient characteristics and clinical data of ocular-surface indexes.

No.	Age	Sex	MD (months)	Eye	Demodex	BUT(s)		TMH(mm)		CFS		Meibomian Gland Morphology							
												CR (%)		TR (%)		GT		GS	
						Pre	Post	Pre	Post	Pre	Post	Pre	Post	Pre	Post	Pre	Post	Pre	Post
1	12	F	36	R	+	3.0	4.6	0.30	0.35	2	1	61.61	63.74	71.28	76.16	5.55	7.19	6.50	6.54
				L	+	3.0	3.2	0.34	0.26	3	1	55.40	59.74	72.98	73.73	6.39	4.78	6.04	6.65
2	6	M	12	R	+	1.6	7.7	0.09	0.15	0	0	38.46	47.08	60.31	78.63	5.14	7.03	5.12	6.16
				L	+	11.3	11.2	0.11	0.12	8	4	22.54	34.62	43.33	69.63	8.99	9.63	5.46	8.42
3	8	F	5	R	+	4.2	4.0	0.19	0.20	0	0	52.42	50.75	64.73	76.67	8.06	6.11	4.17	5.36
				L	+	2.9	6.7	0.21	0.19	0	0	22.91	30.23	28.54	78.94	14.60	21.92	6.15	6.84
4	15	M	5	R	+	5.9	7.1	0.14	0.16	0	0	57.43	59.52	79.82	76.38	14.05	12.32	4.31	3.94
				L	+	3.1	6.8	0.18	0.20	0	0	47.16	62.73	35.17	83.91	6.63	9.02	3.88	3.44
5	15	F	6	R	+	6.7	7.8	0.15	0.25	1	0	45.98	54.87	71.47	71.50	4.41	3.86	4.41	4.49
				L	+	11.3	12.8	0.11	0.22	1	0	44.02	57.52	85.45	85.07	10.59	13.38	4.30	4.75
6	12	F	8	R	-	3.1	3.8	0.15	0.16	7	1	41.05	48.01	68.59	74.16	7.65	7.95	5.27	6.44
				L	-	3.9	3.5	0.13	0.18	5	0	36.13	42.18	84.99	71.15	10.94	12.59	4.60	7.42
7	9	M	24	R	+	3.3	10.1	0.14	0.15	0	0	43.53	43.87	75.08	80.48	6.47	11.20	3.56	3.62
				L	+	3.3	12.0	0.25	0.15	0	0	36.64	48.77	82.57	77.38	7.18	11.05	3.83	4.93
8	11	F	6	R	+	3.4	8.5	0.22	0.25	0	0	59.42	56.53	77.53	79.46	7.90	6.88	6.47	6.58
				L	+	11.7	13.4	0.21	0.23	0	0	45.34	38.43	85.11	81.73	10.69	8.89	3.96	4.69
9	16	F	36	R	-	3.0	7.7	0.24	0.21	0	0	55.12	54.30	76.69	75.87	6.62	7.50	7.09	9.44
				L	-	6.1	4.8	0.15	0.18	0	0	37.89	44.25	80.38	80.89	19.55	10.93	5.82	7.60
10	16	F	48	R	-	5.2	4.9	0.23	0.22	0	1	55.25	60.41	70.36	74.58	7.15	6.39	7.74	5.80
				L	-	2.9	4.2	0.19	0.18	1	1	52.02	52.75	68.47	77.35	9.21	9.16	5.76	6.00
11	16	F	42	R	+	NA[1]	NA[1]	0.14	0.15	0	0	52.80	49.11	75.19	77.68	7.82	6.83	5.92	6.09
				L	+	NA[1]	NA[1]	0.25	0.23	0	0	53.04	62.39	79.53	73.88	9.04	8.95	6.00	7.07
12	16	F	24	R	-	2.7	8.8	0.11	0.15	1	0	32.86	41.96	81.96	72.19	7.38	10.86	7.86	8.60
				L	-	4.0	10.5	0.12	0.15	0	0	48.60	43.05	80.44	82.26	8.30	7.80	9.09	10.24
13	13	M	12	R	-	5.0	9.2	0.35	0.34	0	0	59.47	54.03	75.77	82.32	12.68	14.81	6.65	6.70
				L	-	4.4	5.4	0.30	0.38	0	0	41.48	44.12	81.47	83.35	5.44	13.51	4.10	5.39
14	13	M	6	R	+	15.5	11.8	0.26	0.20	0	0	54.39	58.83	83.82	73.41	9.67	11.60	3.99	5.50
				L	+	9.5	10.1	0.29	0.23	0	0	51.75	60.28	79.72	82.38	8.62	19.76	3.49	5.16
15	7	F	3	R	+	13.6	8.2	0.25	0.22	7	4	16.90	52.58	12.22	36.49	11.67	6.23	4.81	4.80

				L	+	3.1	6.1	0.14	0.19	7	2	8.62	53.82	65.50	79.11	7.31	8.86	4.99	5.83
16	16	M	6	R	+	10.7	9.4	0.22	0.34	2	0	59.11	61.15	72.81	74.43	9.42	8.91	6.38	7.24
				L	+	2.5	1.9	0.16	0.23	1	0	58.66	56.78	71.22	77.57	8.95	10.95	6.09	6.40
17	5	M	1	R	-	3.4	2.2	0.12	0.15	1	0	57.27	56.50	89.40	84.99	6.69	7.13	2.91	3.51

MD, misdiagnosis duration; CR, central gland area ratio; TR, total gland area ratio; GT, gland tortuosity index; GS, gland signal index.

[1] patient No.11 could not obtain BUT data because of corneal irregularities.
